# Theoretical Study on Generation of Multidimensional Focused and Vector Vortex Beams via All-Dielectric Spin-Multiplexed Metasurface

**DOI:** 10.3390/nano12040580

**Published:** 2022-02-09

**Authors:** Yue Liu, Li Chen, Chengxin Zhou, Kuangling Guo, Xiaoyi Wang, Yuhan Hong, Xiangbo Yang, Zhongchao Wei, Hongzhan Liu

**Affiliations:** 1 Guangdong Provincial Key Laboratory of Nanophotonic Functional Materials and Devices, Guangzhou 510006, China; liuyyy_chn@163.com (Y.L.); 2019022102@m.scnu.edu.cn (L.C.); cxzhousss@163.com (C.Z.); klguo99@163.com (K.G.); wxyi19971225@163.com (X.W.); hongyuhan1998@163.com (Y.H.); xbyang@scnu.edu.cn (X.Y.); wzc@scnu.edu.cn (Z.W.); 2 School of Information and Optoelectronic Science and Engineering, South China Normal University, Guangzhou 510006, China

**Keywords:** optical vortex, vector vortex beam, spin-multiplexed, all-dielectric metasurface

## Abstract

The optical vortex (OV) beams characterized by orbital angular momentum (OAM) possess ubiquitous applications in optical communication and nanoparticle manipulation. Particularly, the vortex vector beams are important in classical physics and quantum sciences. Here, based on an all-dielectric transmission metasurface platform, we demonstrate a spin-multiplexed metadevice combining propagation phase and Pancharatnam–Berry (PB) phase. By utilizing a phase-only modulation method, the metadevice can generate spin-dependent and multidimensional focused optical vortex (FOV) under the orthogonally circularly polarized incident light, and it can successfully realize the multiplexed of the above-mentioned FOVs for linearly polarized light. Meanwhile, the superposition of multiple OAM states can also produce vector vortex beams with different modes. Additionally, the evolution process of the electric field intensity profile is presented after the resultant vector vortex beams through a horizontal linear polarization. This work paves an innovative way for generating structured beams, and it provides promising opportunities for advanced applications in optical data storage, optical micromanipulation, and data communication.

## 1. Introduction

Optical vortex (OV) refers to a laser beam carrying the orbital angular momentum (OAM), which is prominently characterized by a helical phase front and doughnut-shaped intensity profile in the plane orthogonal to the light propagation [1]. It can be expressed by a transverse phase structure of exp(*ilφ*), where *l* is the topological charge as any integer and *φ* is the azimuthal angle. Compared to spin angular momentum (SAM) only taking two values of ±*ħ*, the OAM carrying by each photon of the OV beam is *lħ* (where *ħ* is the reduced Planck’s constant) [2]. OAM, as a natural attribute of OV beam, has aroused considerable interest on its unique properties and opens a new esplanade for quantum information [3], optical communications [4], optical tweezers [5], biomedicine [6], and nonlinear optics [7] since it was first proposed by Allen et al. in 1992. For example, OAM with infinite eigenstates can greatly improve the capacity of optical communication systems and is also a potential candidate for high-capacity, high-secure communication systems [8]. In particular, vector vortex beam can be realized by the superposition of multiple OAM states [9] and plays a vital role in optical capture [10], laser processing [11], and optical communication [12]. The traditional approach of generating an OV beam mainly includes spiral phase plates [13], spatial light modulators [14], q-plates [15], computational holography [16], and astigmatic mode converters [17]. The vector vortex beam can be gained by the superposition of multiple spatial beams, phase plates, and spatial light modulators [18]. However, these methods usually require complicated optical systems and bulky optical components, which is not conducive to the development of device miniaturization and integration and further limits the application of OV beams.

Metasurfaces, the intriguing two-dimensional (2D) metamaterials, are composed of ultrathin arrays of metallic or dielectric elements [19,20,21]. Breaking through conventional devices relies on phase accumulation to obtain phase changes, metasurfaces can employ unprecedented modulations on optical phase, amplitude, and polarization by tailoring the local response of meta-atoms. Benefiting from the unique ability and great potential in light manipulation, metasurfaces have led to a burst of studies in metalens [22,23], hologram [24,25], polarization converters [26], and nonlinear optics [27]. In addition, metadevices that generate OV beams and vector vortex beams have been explored and verified [28,29,30,31]. The above devices realize a propagating vortex beam rather than focused optical vortex (FOV), and the tightly FOV beam can form a gradient force to manipulate particles. Thus, the OV generator and lens can be integrated on a simple metasurface to produce FOV [32,33,34,35]. However, most of the work can only gain a single FOV with fixed topological charge, which will restrict the further tunable application of OV beam. In order to break through this limitation, an ingenious method can be used to concentrate independent polarization control on a metadevice. By changing the polarization state of incident light, multiple FOVs can be generated based on a single-layer metasurface. A series of measures can be taken to realize the spin-multiplexed metasurface; for example, two orthogonally linearly polarized (LP) lights can be individually regulated by the propagation phase. However, this method requires a lot of parameters scanning to match the desired phases, which increases the complexity and may also cause crosstalk [21,36]. Furthermore, the Pancharatnam–Berry (PB) phase is widely applied due to its flexible phase modulation mechanism and excellent polarization multiplexed capability for circularly polarized light, and many spin-dependent vortex metalenses based on pure PB phase are designed. Unfortunately, the theoretically maximum efficiency is 50% when LP light is incident on the metasurface, and the maximum efficiency can only reach 25% for each spin state, hindering the development of spin-multiplexed focused vortex metalens due to the inevitable problem of lower efficiency [37,38]. Recently, Capasso’s group manipulated any two orthogonal polarization states by combining the propagation phase and PB phase to obtain high-efficiency polarization multiplexed metasurfaces [39]. This novel method greatly expands the application scope of metasurface polarization optics and paves an innovative way to generate high-efficiency, multichannel, and multidimensional FOVs and vector vortex beams. Using a kind of dielectric metasurface composed of staggered arrangements of anisotropic and isotropic meta-atoms, Zheng et al. experimentally demonstrated polarization-controllable superpositions of OAM states in the terahertz band by combining the propagation phase and PB phase [40]. Xu et al. produced two types of polarization-dependent metasurfaces and successfully presented the terahertz cylinder vector beam and vector Bessel beam [41]. Wang et al. numerically studied high-efficiency spin-related vortex metalenses at the mid-infrared band through engineering both the propagation phase and PB phase subtly [42]. Based on arranging the geometric parameters of all-dielectric meta-atoms, Liu et al. gained stable and high-quality perfect Poincaré beams using spin-multiplexed metasurface for visible light [43]. However, researchers are dedicated to produce spin-multiplexed different beams at one focal plane. There have been no reports on generating multidimensional spin-multiplexed FOVs and vector vortex beams via the proposed method.

**Figure 1:**
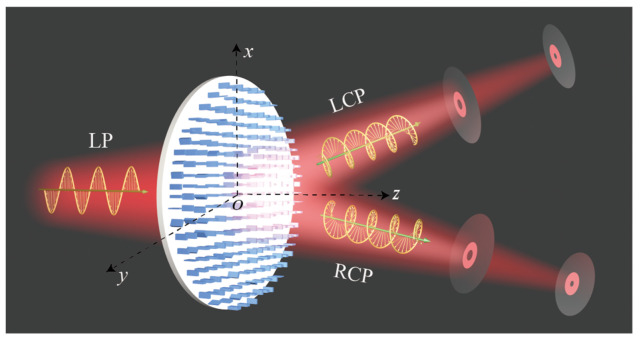
Schematic diagram of multidimensional focused vortex beams generated based on the dielectric spin-multiplexed metasurface.

In this paper, we demonstrate an all-dielectric spin-multiplexed metasurface integrating OV generators and lens by combining the propagation phase and PB phase at 1500 nm. As shown in Figure 1, the designed metasurface can provide two arbitrary and multidimensional FOVs under left-handed circularly polarized (LCP) and right-handed circularly polarized (RCP) illumination. When a LP light shines on the metasurface, all of the aforementioned spin-dependent FOVs can be observed simultaneously, which successfully achieves the multiplexed of the FOVs. Unlike previous studies that could only produce polarization insensitive multichannel FOVs at a specific focal plane [32], it is also different from some reports that can only obtain multichannel vortex beams under a particularly circularly polarized light [44,45]. Surprisingly, the problem of low efficiency in generating spin-multiplexed FOV beams using pure PB phase is also addressed [37]. It is worth mentioning that the number of FOV, the corresponding topological charge, and the focal plane position can be adjusted flexibly according to different requirements. Additionally, the superposition of OAM states at different focal planes can also be utilized to acquire multidimensional vector vortex beams. By controlling the polarization state of incident light, we show the electric field intensity profiles of the generated vector vortex beams passing through a horizontal linear polarizer. We numerically studied spin-multiplexed metasurfaces to generate multidimensional FOVs and vector vortex beams, the results greatly promote the OAM communication system, multidimensional information storage, and applications of integrated optics. Similarly, we also provide design guidelines for a metasurface capable of generating and multiplexing multiple tailored vortex beams.

## 2. Materials and Methods

As we know, when an LP light is normally incident on the birefringent nanopillars, the input and output electric field can be expressed as [21]:

(1) $$\left[\begin{array}{c} E_{x}^{o}\\ E_{y}^{o} \end{array}\right]=M(x,y)\left[\begin{array}{c} E_{x}^{i}\\ E_{y}^{i} \end{array}\right]=R(-\theta )\left[\begin{array}{cc} e^{i\phi_{x}} & 0\\ 0 & e^{i\phi_{y}} \end{array}\right]R(\theta )\left[\begin{array}{c} E_{x}^{i}\\ E_{y}^{i} \end{array}\right]$$

where $E_{x}^{i}$ and $E_{y}^{i}$ indicate the *x* and *y* components of the input electric field, $E_{x}^{o}$ and $E_{y}^{o}$ are the *x* and *y* components of the output electric field, respectively. *M*(*x, y*) is the transmission matrix, *R* indicates a 2 × 2 rotation matrix, and *θ* is the rotation angle of nanopillars relative to the reference coordinate system. $\phi_{x}$ and $\phi_{y}$ denote the phase retardation of nanopillars with respect to the *x*-linearly polarized (XLP) and *y*-linearly polarized (YLP) light, respectively.

The metasurface can implement independent phase modulation for LCP and RCP to generate FOVs. For example, the metasurface can achieve phase modulation of $\varphi_{1}$(*x, y*) for LCP incident light. When the incident light is converted into RCP, the metasurface can employ $\varphi_{2}$(*x, y*). It is known that the metasurface can be represented by a transmission matrix *M*(*x*, *y*), which can take the form [39,43]:

(2) $$M(x,y)|LCP\rangle =\delta_{1}\exp\left[i\varphi_{1}(x,y)\right]|RCP\rangle$$

(3) $$M(x,y)|RCP\rangle =\delta_{2}\exp\left[i\varphi_{2}(x,y)\right]|LCP\rangle$$

where $|LCP\rangle =\left[\begin{array}{c} 1\\ i \end{array}\right]$ and $|RCP\rangle =\left[\begin{array}{c} 1\\ -i \end{array}\right]$. $\delta_{1}$ and $\delta_{2}$ denote the conversion factors, because the diffraction efficiency is lower than 100%. The matrix inversion of Equations (2) and (3) results in:

(4) $$M(x,y)=\left[\begin{array}{cc} \delta_{1}\exp\left[i\varphi_{1}(x,y)\right] & \delta_{2}\exp\left[i\varphi_{2}(x,y)\right]\\ -i\delta_{1}\exp\left[i\varphi_{1}(x,y)\right] & i\delta_{2}\exp\left[i\varphi_{2}(x,y)\right] \end{array}\right]{\left[\begin{array}{cc} 1 & 1\\ i & -i \end{array}\right]}^{-1}.\;$$

Then, Equations (1) and (4) can be used to calculate the desired phase shifts and rotation angle:

(5) $$\phi_{x}(x,y)=\left[\varphi_{1}(x,y)+\varphi_{2}(x,y)\right]/2$$

(6) $$\phi_{y}(x,y)=\left[\varphi_{1}(x,y)+\varphi_{2}(x,y)\right]/2-\pi$$

(7) $$\theta (x,y)=\left[\varphi_{1}(x,y)-\varphi_{2}(x,y)\right]/4.$$

**Figure 2:**
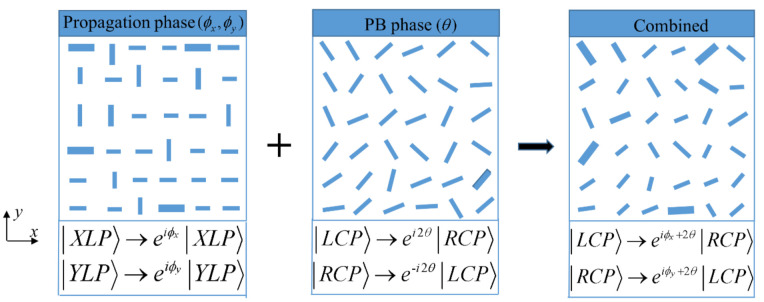
The relationship between propagation, PB, and combined phases. The combined phase is obtained by simultaneously adjusting the propagation phase (only changing the size of meta-atoms) and the PB phase (only changing the rotation angle of meta-atoms).

It can be seen from Equations (5)–(7) that the phase difference between $\phi_{x}$ and $\phi_{y}$ is π acting as a half-wave plate and the phase modulation of the metasurface is mainly determined by $\phi_{x}$ and *θ*, corresponding to the propagation phase and PB phase, respectively. Figure 2 shows the relationship between propagation, PB, and combined phases. The propagation phase is controlled by the dimension of nanopillars, while the PB phase is related to the rotation angle of nanopillars. The simultaneous changes in geometry size and rotation angle are equal to combining propagation phase and PB phase. Thus, a single-layer metasurface encoded unrelated phase profiles $\varphi_{1}$(*x*, *y*) and $\varphi_{2}$(*x*, *y*) for LCP and RCP light, respectively, can generate spin-multiplexed FOVs. Specifically, the metasurface can produce a FOV beam with topological charge of *l_m_* under LCP illumination. That is, the metasurface implements transformation $|LCP\rangle \to |FOV_{R},l_{m}\rangle$. Due to the introduction of the PB phase, the transmitted beam FOV has flipped handedness compared with the incident light. Similarly, the metasurface performs conversion $|RCP\rangle \to |\langle FOV_{L},l_{n}|\rangle$ when the incident light is switched from LCP to RCP.

**Figure 3:**
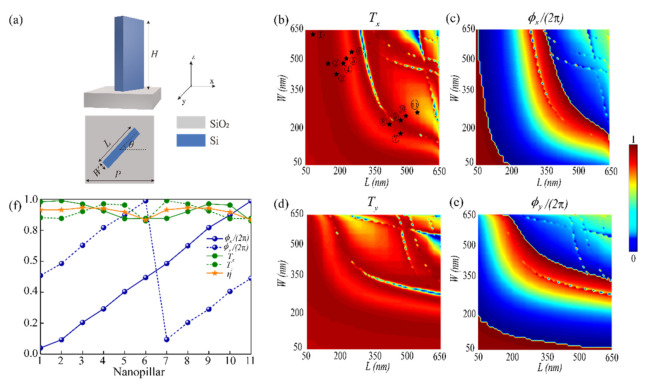
(**a**) Perspective and top views of the meta-atom which consists of a rectangular a-Si nanopillar patterned on the square SiO_2_ substrate. (**b**,**c**) The transmission coefficients and phase shifts for XLP illumination. (**d**,**e**) The transmission coefficients and phase shifts under YLP incident light. (**f**) The corresponding transmission coefficients, phase shifts, and polarization conversion efficiencies of all selected 11 nanopillars.

In order to efficiently modulate the propagation phase and PB phase, the units constituting the metasurface are designed strictly. A series of subwavelength nanopillars is governed to provide the desired phase shifts ($\phi_{x}$, $\phi_{y}$) covering the entire 2π range and satisfy the rotation angle *θ* of any point (*x*, *y*) on the metasurface. Figure 3a presents a meta-atom composed of rectangular amorphous silicon (a-Si) nanopillar (length *L*, width *W*, and height *H*) embedded on the square SiO_2_ substrate with a lattice constant *P*. The refractive indexes of the rectangular nanopillar and square substrate are 3.48 and 1.48, respectively. Due to the angular asymmetry of the unit geometry, it can be considered as a rectangular waveguide exhibiting birefringence property. The phase delays $\phi_{x}$ and $\phi_{y}$ of the meta-atom along the *x*- and *y*- axes can be achieved by varying *L* and *W*. To investigate the transmission properties of rectangular nanopillars, all simulations are performed based on 3D-finite-difference-time-domain (FDTD). The height *H* of the rectangular a-Si nanopillar is set as 1000 nm, and the lattice constant is *P* = 650 nm. The length (*L*) and width (*W*) are swept from 50 to 650 nm with an interval of 7.5 nm. By scanning the nanopillars with different geometrical parameters, we calculate the phase shifts and transmission coefficients of rectangular a-Si nanopillars for XLP and YLP light at the design wavelength of *λ* = 1500 nm. The incident light is plane wave and propagates along the +*z* direction. Periodic boundary conditions are applied in the *x* and *y* directions, and perfectly matched layers (PMLs) are implemented in the *z* direction. The mesh grids are set as 30 nm × 30 nm × 50 nm. Figure 3b,c describe the transmission coefficient $T_{x}$ and phase shift $\phi_{x}$ as a function of the nanopillar size (*L*, *W*) upon the XLP light. For YLP incident light, the transmission coefficient $T_{y}$ and phase shift $\phi_{y}$ can be regarded as the transposition of $T_{x}$ and $\phi_{x}$, as shown in Figure 3d,e. It can be seen from Figure 3b–e that the phase delays can almost span over the full 2π range, so any phase combination ($\phi_{x}$, $\phi_{y}$) can be gained by choosing the nanopillars size (*L*, *W*) reasonably. A set of 11 nanopillars (black pentagrams in Figure 3b) with an interval of 2π/11 are optimized, and the corresponding transmission coefficients, phase shifts, and polarization conversion efficiencies are shown in Figure 3f. The transmission coefficients ($T_{x}$, $T_{y}$) of 11 nanopillars remain above 85%, and the phase difference between $\phi_{x}$ and $\phi_{y}$ approaches *π*. At the same time, these nanopillars can achieve high-efficiency polarization conversion. The polarization conversion efficiency is defined at the focal plane by $\eta ={\left|E_{RCP}\right|}^{2}/({\left|E_{RCP}\right|}^{2}+{\left|E_{LCP}\right|}^{2})$. So these selected nanopillars can be regarded as quasi-perfect half-wave plates. In conclusion, a metasurface composed of 11 nanopillars can implement independent and arbitrary phase modulation for LCP and RCP light.

## 3. Results and Discussion

### 3.1. Generation of Multidimensional FOVs

**Figure 4:**
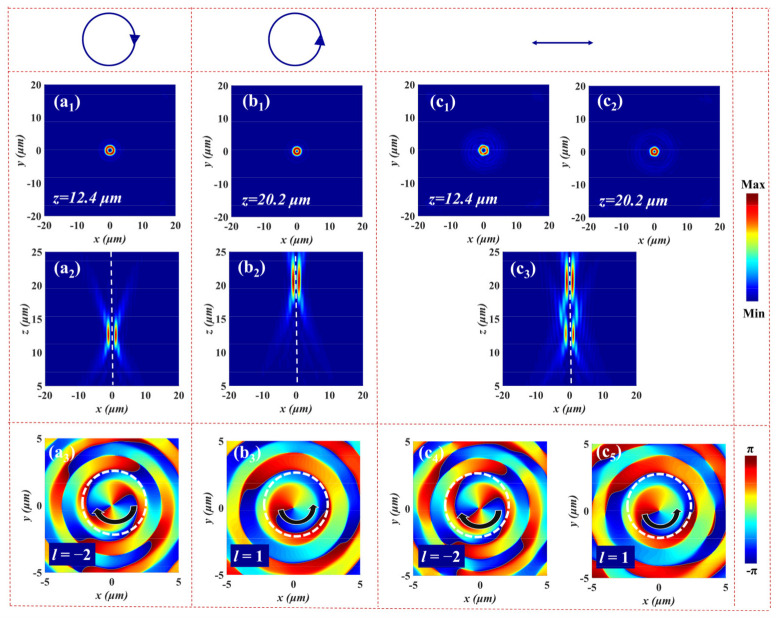
The simulated electric field intensity distributions of the FOV at the focal plane for (**a_1_**) LCP, (**b_1_**) RCP, and (**c_1_**,**c_2_**) XLP illumination. The electric field intensity profiles of the FOV in the *x-z* plane upon the illumination with (**a_2_**) LCP, (**b_2_**) RCP, and (**c_3_**) XLP input light. The corresponding phase distributions in the *x*-*y* plane at the focal plane under (**a_3_**) LCP, (**b_3_**) RCP, and (**c_4_**,**c_5_**) XLP incident light.

According to the above analysis, a spin-multiplexed metasurface can be realized by combining the propagation phase and PB phase. Designing and arranging meta-atoms elaborately, the metadevice has the ability to generate arbitrary, spin-dependent, and multidimensional FOVs under LCP and RCP incidence. Therefore, the phase profiles of metasurface are governed by [42]:

(8) $$\varphi_{LCP}(x,y)=-\frac{2\pi }{\lambda }(\sqrt{x^{2}+y^{2}+f_{L}^{2}{}^{}}-f_{L}^{})+l_{m}\cdot \;\arctan(y/x)$$

(9) $$\varphi_{RCP}(x,y)=-\frac{2\pi }{\lambda }(\sqrt{x^{2}+y^{2}+f_{R}^{2}{}^{}}-f_{R}^{})+l_{n}\cdot \;\arctan(y/x)$$

where *λ* is the working wavelength, *f_L_* and *f_R_* are the focal lengths of LCP and RCP incident light along the *z* direction, and *l_m_* and *l_n_* represent the topological charges of FOVs for LCP and RCP light, respectively. Specifically, λ = 1.55 μm, *f_L_* = 12 μm, *f_R_* = 20 μm, *l_m_* = −2, and *l_n_* = 1. The focused vortex metalens composed of 61 × 61 micropillars is employed to generate spin-dependent FOVs at different focal planes under the orthogonally circularly polarized light. The diameter of vortex metalens is about 39.5 μm (D = 39 μm). To reduce the simulation time and ensure accuracy, we establish a 3D FDTD simulation model with a simulation region of 20 µm × 20 µm × 2 µm, and a mesh size of 30 nm × 30 nm × 50 nm is employed in the multifunctional metadevices. The *PML* boundary conditions are applied along all the three axes for the simulations. Here, we can get the required far-field information by projecting the near-field data to the far-field. The numerical simulation results are shown in Figure 4. Figure 4a_1_ displays the donut-shaped intensity distribution of electric field at the focal plane for LCP illumination. The electric field intensity profile of the *x-z* plane is given in Figure 4a_2_. It can be seen that the FOV is located at *z* = 12.4 μm, which is close to the preset value (*f_L_* = 12 μm). Figure 4a_3_ demonstrates the phase distribution of the FOV along the *x-y* plane at *z* = 12.4 μm, where the number and direction of the spiral represent the number and sign of topological charge, implying that a FOV with topological charge *l* = −2 is generated under LCP incident light. The focusing efficiency of the transmitted beam FOV is 62%. (The focusing efficiency is given by the ratio of the focusing power to the total incident power). Correspondingly, Figure 4b_1_–b_3_ show the electric field intensity distributions and phase profile of the output beam FOV under RCP incidence. That is, a FOV with topological charge of *l* = 1 is generated at *z* = 20.2 μm with a focusing efficiency of 65% for RCP light. According to the calculation of the numerical aperture (NA) = sin[tan^−1^(*D*/2*Fi*)], the NA of the two FOVs are 0.844 and 0.695 for LCP and RCP incident light. It can be found from Figure 4a_1_–b_3_ that the vortex metalens can independently modulate the phases of LCP and RCP. When the incident light is converted into XLP, two FOVs can be observed at *z* = 12.4 μm and *z* = 20.2 μm, respectively, as shown in Figure 4c_1_–c_5_, which successfully realizes the multiplexed spin-dependent FOVs.

**Figure 5:**
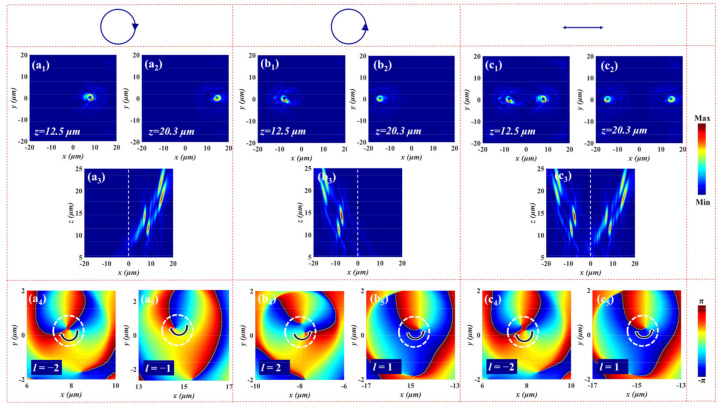
The simulated electric field intensity distributions of FOVs at the focal plane for (**a_1_**,**a_2_**) LCP, (**b_1_**,**b_2_**) RCP, and (**c_1_**,**c_2_**) XLP illumination. The electric field intensity profiles of FOVs in the *x-z* plane upon the illumination with (**a_3_**) LCP, (**b_3_**) RCP, and (**c_3_**) XLP input light. The corresponding phase distributions at the focal plane in the *x*-*y* plane under (**a_4_**,**a_5_**) LCP, (**b_4_**,**b_5_**) RCP, and (**c_4_**,**c_5_**) XLP incident light.

To prove the robustness of our proposed method, we can also generate multidimensional FOVs under LCP and RCP illumination. The required phase profiles are expressed as:

(10) $$\varphi_{{}_{LCP}}^{}(x,y)=\arg(\sum_{M}\exp(i\varphi_{{}_{L}}^{M}))$$

(11) $$\varphi_{{}_{RCP}}^{}(x,y)=\arg(\sum_{N}\exp(i\varphi_{{}_{R}}^{N}))$$

where $\varphi_{{}_{L}}^{M}(x,y)=-\frac{2\pi }{\lambda }(\sqrt{{\left(x-x_{L}^{M}\right)}^{2}+{\left(y-y_{L}^{M}\right)}^{2}+{\left(f_{L}^{M}\right)}^{2}}-f_{L}^{M})+l_{L}^{M}\cdot \;\arctan(y/x)$ and $\varphi_{{}_{R}}^{N}(x,y)=-\frac{2\pi }{\lambda }(\sqrt{{\left(x-x_{{}_{R}}^{N}\right)}^{2}+{\left(y-y_{{}_{R}}^{N}\right)}^{2}+{\left(f_{R}^{N}\right)}^{2}}-f_{R}^{N})+l_{R}^{N}\cdot \;\arctan(y/x)$.$\left(x_{L},y_{L},f_{L}\right)$ and $\left(x_{R},y_{R},f_{R}\right)$ are the focal position of FOVs for the normal incidence of LCP and RCP light. *M* and *N* denote the number of FOVs. The radius of FOV is taken into account in the simulation to avoid interference affecting the output beam performance. The designed parameters are M=N=1,2, $f_{L}^{1}=f_{R}^{1}=12\;\mu m$, $f_{L}^{2}=f_{R}^{2}=20\;\mu m$, $x_{L}^{1}=-x_{R}^{1}=8\;\mu m$, $x_{L}^{2}=-x_{R}^{2}=15\;\mu m$, $l_{L}^{1}=-l_{R}^{1}=-2$, $l_{L}^{2}=-l_{R}^{2}=-1$, and $y_{L}^{1}=y_{L}^{2}=y_{R}^{1}=y_{R}^{2}=0$. Employing the FDTD method, when the incident light is LCP, two FOVs can be generated and off-axis distributed at the right half space (7.8, 0, 12.5) and (14.7, 0, 20.3) from Figure 5a_1_–a_3_. The corresponding focusing efficiencies are 27.4% and 16.2%, respectively. Figure 5a_4_,a_5_ show the phase profiles of the produced FOVs with topological charges *l* = −2 and *l* = −1 in the *x*-*y* plane. Similarly, two FOVs with focusing efficiencies of 35.3% and 20.1% are observed in the left half space, and the focusing positions are (−7.8, 0, 12.5) and (−14.7, 0, 20.3), respectively (see Figure 5b_1_–b_3_). The phase distributions of FOVs with the topological charges *l* = 2 and *l* = 1 along the *x*-*y* plane for RCP are presented in Figure 5b_4_,b_5_. Therefore, the metasurface can implement spin-dependent and multidimensional FOVs under LCP and RCP incident light. From the Figure 5c_1_–c_3_, we can observe four FOVs mentioned above under XLP incident light. In order to analyze the phase of output beams intuitively, only the phase profiles of FOVs at (7.8, 0, 12.5) and (−14.7, 0, 20.3) are given here (see Figure 5c_4_,c_5_). Additionally, some deviations (the inconsistency of the focus position and the imperfect phase distribution) between the simulation results and preset values can be attributed to the discrete phase profile of the metasurface for approximation of the continuous phase distribution, which leads to the optical response of selected nanopillars not exactly matching the required phases. However, these small deviations will not fundamentally affect the metadevice to generate multidimensional spin-multiplexed FOVs.

### 3.2. Generation of Multidimensional Vector Vortex Beams

**Figure 6:**
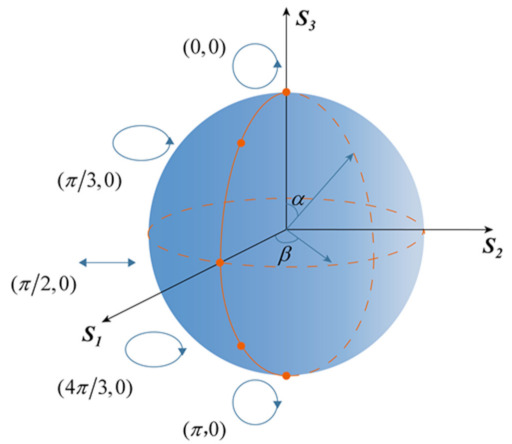
Schematic view of a vector vortex beam with space-dependent polarization distribution and phase profile on the HyOPS.

Notably, in addition to generating multidimensional spin-multiplexed FOVs, the metasurface can also successfully realize the multiplexed vector vortex beams through the superposition of OAM states. A vector vortex beam can be obtained by the linear superposition of two orthogonally circularly polarized FOVs, which can be expressed as [46]:

(12) $$|E\rangle =\cos(\alpha /2)e^{i\beta /2}|FOV_{R},l_{m}\rangle +\sin(\alpha /2)e^{-i\beta /2}|FOV_{L},l_{n}\rangle$$

where $|FOV_{R},l_{m}\rangle$ and $|FOV_{L},l_{n}\rangle$ are the RCP and LCP FOV with different topological charge numbers of *l_m_* and *l_n_*, respectively. *cos*(*α*/2) and *sin*(*α*/2) denote the amplitude of RCP and LCP FOV, and *β* is the relative phase difference between them. To more clearly explain the implementation of the vector vortex beam, we introduce a hybrid-order Poincaré Sphere (HyOPS) in Figure 6. $|E\rangle$ in Equation (12) describes any point with spherical coordinates (*α, β*) on the HyOPS, and each point represents a vector vortex beam with space-dependent polarization distribution and phase profile, where the polarization distribution can be determined by the polarization order *p* = (*l_m_* − *l_n_*)/2, and the phase profile can be characterized by the topological Pancharatnam charge *l_p_* = (*l_m_* + *l_n_*)/2. Specifically, five points are selected on the HyOPS surface, which indicates five polarization states of the incident light, including LCP, left-handed elliptically polarized (LEP), LP, right-handed elliptically polarized (REP), and RCP, as shown in Figure 6. Among them, only a single FOV is generated at the specific focal plane for LCP and RCP. In particular, the vector vortex beam will be produced by the superposition of FOVs with equal power when the LP impinges on the metasurface.

**Figure 7:**
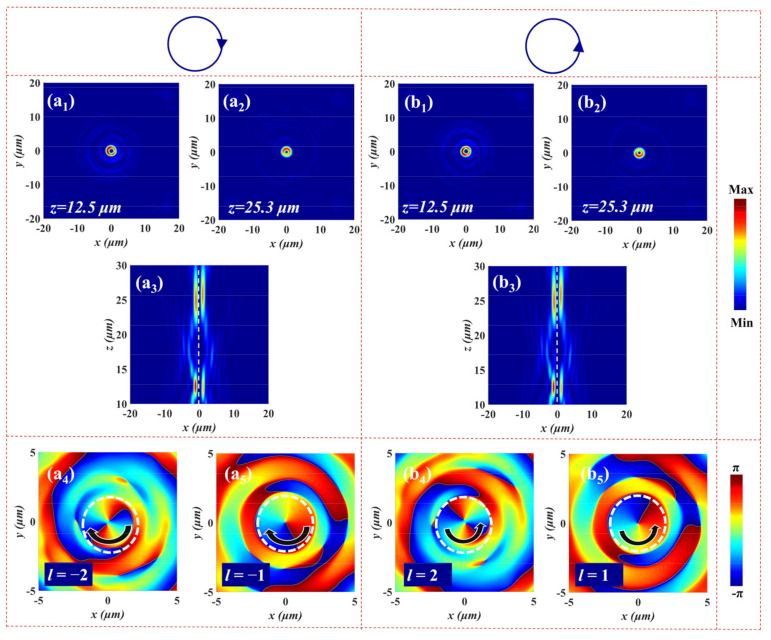
The simulated electric field intensity distributions of FOVs at the focal plane for (**a_1_**,**a_2_**) LCP and (**b_1_**,**b_2_**) RCP. The electric field intensity profiles of FOVs in the *x-z* plane upon the illumination with (**a_3_**) LCP and (**b_3_**) RCP. The corresponding phase distributions in the *x-z* plane at the focal plane under (**a_4_**,**a_5_**) LCP and (**b_4_**,**b_5_**) RCP.

Based on the above principle, we demonstrate a spin-multiplexed metasurface that can simultaneously realize different modes of vector vortex beams. The required phase distributions are the same as in Equations (10) and (11); here, the designed parameters are M=N=1,2, $x_{L}^{1}=x_{L}^{2}=x_{R}^{1}=x_{R}^{2}=0$, $y_{L}^{1}=y_{L}^{2}=y_{R}^{1}=y_{R}^{2}=0$, $f_{L}^{1}=f_{R}^{1}=12\;\mu m$, $f_{L}^{2}=f_{R}^{2}=25\;\mu m$, $l_{L}^{1}=-l_{R}^{1}=-2$, and $l_{L}^{2}=-l_{R}^{2}=-1$. Figure 7 presents the electric field distributions (*x*-*y* plane and *x*-*z* plane) and phase profiles (*x*-*y* plane) of the generated FOVs for LCP and RCP illumination. Under LCP incident light, two FOVs are observed at (0, 0, 12.5) and (0, 0, 25.3) with focusing efficiencies of 41.2% and 35.3%, respectively (see Figure 7a_1_–a_3_). The corresponding phase distributions along the *x*-*y* plane are shown in Figure 7a_4_,a_5_. When the incident light is converted into RCP, two FOVs with focusing efficiencies of 41.8% and 35.4% can be seen at the same positions from Figure 7b_1_–b_3_. The NA of the two beams are 0.845 (*z* = 12.5 μm) and 0.611 (*z* = 25.3 μm), respectively. It is noteworthy that the sign of topological charge of the output beam FOV generated by RCP is opposite to that produced by LCP light in Figure 7b_4_,b_5_. We can conclude that FOVs with a topological charge of *l* = ∓2 and *l* = ∓1 are gained at *z* = 12.5 μm and *z* = 25.3 μm under LCP and RCP incident light, respectively. When a LP light is incident onto the metasurface, the equal components of $|FOV_{R},l_{-2}\rangle$ and $|FOV_{L},l_{2}\rangle$ will further superimpose at the focal plane *z* = 12.5 μm to produce a vector vortex beam with the polarization order *p* = (−2–2)/2 = −2. Similarly, the linear combination of equal-weighted $|FOV_{R},l_{-1}\rangle$ and $|FOV_{L},l_{1}\rangle$ can generate a radial vector vortex beam with the polarization order *p* = (−1–1)/2 = −1 at *z* = 25.3 μm for XLP light.

To better diagnose the resultant vector vortex beam, it can be characterized by a linear polarizer with *χ* orientation angle from the *x*-axis. The transmitted intensity of the vector vortex beam through a linear polarizer is governed by:

(13) $$T_{LP}={\left|\left[\begin{array}{cc} \cos^{2}\chi & \cos\chi \sin\chi \\ \cos\chi \sin\chi & \sin^{2}\chi \end{array}\right]•|U_{N}\rangle \right|}^{2}.$$

**Figure 8:**
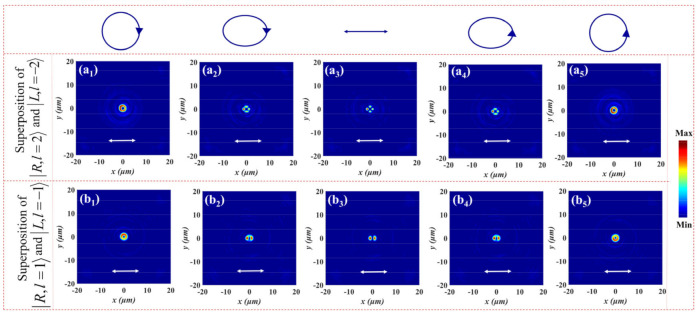
The electric field intensity profiles of generated beams corresponding to five po-larized states of the HyOPS after transmission through a horizontal linear polarizer at different focal planes for (**a_1_**,**b_1_**) LCP, (**a_2_**,**b_2_**) LEP, (**a_3_**,**b_3_**) XLP, (**a_4_**,**b_4_**) REP, and (**a_5_**,**b_5_**) RCP illumination. The analyzing polarizer is depicted by the white double arrow.

The above-mentioned polarization order *p* represents the number of polarization rotations per round trip. Therefore, the lobe number of the pattern after the generated vortex beams through a horizontal linear polarizer can be deduced as 2|*p*|. Figure 8 is the electric field intensity profiles of generated beams corresponding to the five points of the HyOPS after transmission through a horizontal linear polarizer depicted by the white double arrow at different focal planes. By continuously changing the polarization state of incident light, the evolution of light intensity can be explored clearly. As analyzed above, only a FOV is generated at *z* = 12.5 μm and *z* = 25.3 μm under LCP and RCP illumination, respectively. The light intensity remains in a donut-shaped distribution after a horizontal linear polarizer, and the annular radius increases with the increase of topological charge, as shown in Figure 8a_1_,a_5_,b_1_,b_5_. Figure 8a_3_ shows that a vector vortex beam can be realized due to the superposition of FOVs at *z* = 12.5 μm for XLP light. The transmitted beam presents a pretty petal-like pattern through the horizontal linear polarizer, and the total number of lobes of the pattern is equal to $2\left|p\right|=4$. In the case of LEP and REP light, the gap between the intensity decreases due to the non-equal superposition of FOVs (see Figure 8a_2_,a_3_). Similarly, it can be seen from Figure 8b_1_,b_5_ that both LCP and RCP incident light can generate annular light intensity distributions at *z* = 25.3 μm. The intensity profiles exhibit two petals due to the polarization order $2\left|p\right|=2$ for LEP and REP light from Figure 8b_2_,b_4_, and a gap between the two petals becomes more clear under XLP illumination (see Figure 8b_3_). Thus, the multidimensional vector vortex beams with different modes can be implemented by a metasurface with phase-only modulation, which provides more opportunities and possibilities for high-capacity data transmission and communication. In addition, different polarized light is normally incident onto the metasurface. The introduction of angular-multiplexed is considered in further studies to realize multifunctional integrated and tunable metadevices.

## 4. Conclusions

In conclusion, we demonstrate the spin-multiplexed vortex metalens by combining both propagation phase and PB phase via all-dielectric metasurface. Through phase-only modulation, single or multiple spin-dependent, arbitrary, and multidimensional FOVs can be generated at different focal planes for LCP and RCP light. While upon the illumination with XLP input light, the multiplexed of FOVs can be realized successfully. In addition, a polarization-controllable metasurface is designed based on the OAM states superposition, and diverse modes of vector vortex beams are displayed at different focal planes. Meanwhile, five points on the HyPOS are selected to show the evolution process of the intensity profiles after the OAM superposition states through a horizontal linear polarizer under the different polarization states of the incident light. This work provides a new simple and effective method for modulating the polarization and phase simultaneously. The alliance between polarization multiplexed and multichannel beams further promotes the development of equipment miniaturization and integration, and it also exhibits a more convenient solution to generate structural beams, which also has a profound impact on light capture and data communication.

## Data Availability

No new data were created or analyzed in this study. Data sharing is not applicable to this article.
